# Semi-supervised prediction of protein interaction sites from unlabeled sample information

**DOI:** 10.1186/s12859-019-3274-7

**Published:** 2019-12-24

**Authors:** Ye Wang, Changqing Mei, Yuming Zhou, Yan Wang, Chunhou Zheng, Xiao Zhen, Yan Xiong, Peng Chen, Jun Zhang, Bing Wang

**Affiliations:** 1 School of Electrical and Information Engineering, Anhui University of Technology, Maanshan, 243002 Anhui China; 2 Co-Innovation Center for Information Supply & Assurance Technology, Anhui University, Hefei, 230601 Anhui China; 3 School of Computer Science and Technology, Anhui University of Technology, Maanshan, 243002 Anhui China; 4 School of Computer Science and Technology, University of Science & Technology, Hefei, 230026 Anhui China; 5 Institute of Health Sciences, Anhui University, Hefei, 230601 Anhui China; 6 College of Electrical Engineering and Automation, Anhui University, Hefei, 230601 Anhui China

**Keywords:** Protein interaction site, Unlabeled information, Conservative feature, Semi-supervised support vector machine

## Abstract

**Background:**

The recognition of protein interaction sites is of great significance in many biological processes, signaling pathways and drug designs. However, most sites on protein sequences cannot be defined as interface or non-interface sites because only a small part of protein interactions had been identified, which will cause the lack of prediction accuracy and generalization ability of predictors in protein interaction sites prediction. Therefore, it is necessary to effectively improve prediction performance of protein interaction sites using large amounts of unlabeled data together with small amounts of labeled data and background knowledge today.

**Results:**

In this work, three semi-supervised support vector machine–based methods are proposed to improve the performance in the protein interaction sites prediction, in which the information of unlabeled protein sites can be involved. Herein, five features related with the evolutionary conservation of amino acids are extracted from HSSP database and Consurf Sever, i.e., residue spatial sequence spectrum, residue sequence information entropy and relative entropy, residue sequence conserved weight and residual Base evolution rate, to represent the residues within the protein sequence. Then three predictors are built for identifying the interface residues from protein surface using three types of semi-supervised support vector machine algorithms.

**Conclusion:**

The experimental results demonstrated that the semi-supervised approaches can effectively improve prediction performance of protein interaction sites when unlabeled information is involved into the predictors and one of them can achieve the best prediction performance, i.e., the accuracy of 70.7%, the sensitivity of 62.67% and the specificity of 78.72%, respectively. With comparison to the existing studies, the semi-supervised models show the improvement of the predication performance.

## Background

Protein-protein interactions (PPIs) are involved in various life activities, such as metabolism and signal transduction, gene transcription, protein translation, modification and localization, and are also closely related to disease production [1–9]. However, PPI varies from cell to cell and from time to time, which poses a challenge to the studies of them.

Due to the rapid development of machine learning methods, many classical methods, such as Bayesian, support vector machine (SVM), and artificial neural networks, have been used to predict protein interaction sites [10–19]. Sprinzak et al. used the correlated sequence-signatures as identifiers for the interacting protein which can significantly reduce the search space and implement a directional experiment interactive screen and achieved high quality experimental results [5]. Bock et al. proposed a phylogenetic bootstrapping algorithm which suggests traversal of a phenogram, interleaving rounds of computation and experiment, to develop a knowledge base of protein interactions in genetically-similar organisms [6]. Enright et al. developed the hydrophobic free energy functions with the fusion detection based on sequence analysis and trained a SVM learning system to recognize and predict interactions based solely on primary structure and associated physicochemical properties, and the overall performance of the classifier has been significantly improved [20]. Chen et al. proposed a radial basis function neural networks optimized by the particle swarm optimization algorithm to predict protein interaction sites [2]. Wang et al. presented a SVM based algorithm to identify protein-protein interactions sites on the residues level by incorporating residues spatial sequence profile and evolution rate [21]. Wang et al. in another work implemented a dataset reconstruction strategy by using manifold learning under a hypothesis that the interaction and non-interaction sites have different inherent structure manifolds [13, 22]. Although these methods have driven advances in PPI research, there is still a problem that a lot of interactions cannot be tagged from experiments, and only a small part of labeled samples can be used for model training in the prediction of PPI sites, which will make it difficult for the well-trained learning systems to have strong generalization ability [23].

Therefore, this paper proposed three semi-supervised machine learning-based computational models to address the problem that the information of a large number of unlabeled samples can be utilized effectively to improve the performance of protein interaction site prediction when only a few of labeled samples can be available. Firstly, five evolutionary conserved features of amino acids based on multiple sequence alignments are extracted, i.e., the spatial sequence spectrum of residues, sequence information entropy, relative entropy, conservative weight and residue evolution rate. Then three semi-supervised learning methods are proposed, i.e., the self-balancing semi-supervised support vector machine based on multi-core learning (Means3vm-mkl), the iterative-based label average self-training semi-supervised support vector machine (Means3vm-iter) and the safe semi-supervised support vector machine (S4VM), to build the prediction model for identification of protein interaction sites [24–26]. The experimental results demonstrated the superiority of our proposed methods, such as the prediction accuracy of 0.707 for S4VM model, with comparison to the existing supervised and other approaches.

## Results

In this work, three semi-supervised SVM algorithms, i.e., Means3vm-mkl, Means3vm-mkl, and S4VM, have been applied for the prediction of protein interaction sites from protein sequences. Compared to the traditional supervised SVM, semi-supervised models can effectively use the information from both of labeled and unlabeled samples. A popular software of support vector classification, Libsvm, is adopted in this work, where the empirically optimal parameters are used, such as *C*_1_ is 1, *C*_2_ equals 0.1, and the maximum number of generations is 50. To validate the effectiveness of the proposed models, a 5-fold cross-validation technique, and an original residue data set with 91 protein chains are used to evaluate the prediction performance of the proposed models. Herein, 2299 interface residues drawn from the definition of interaction sites can be used for the construction of the three semi-supervised SVM models.

**Fig. 1:**
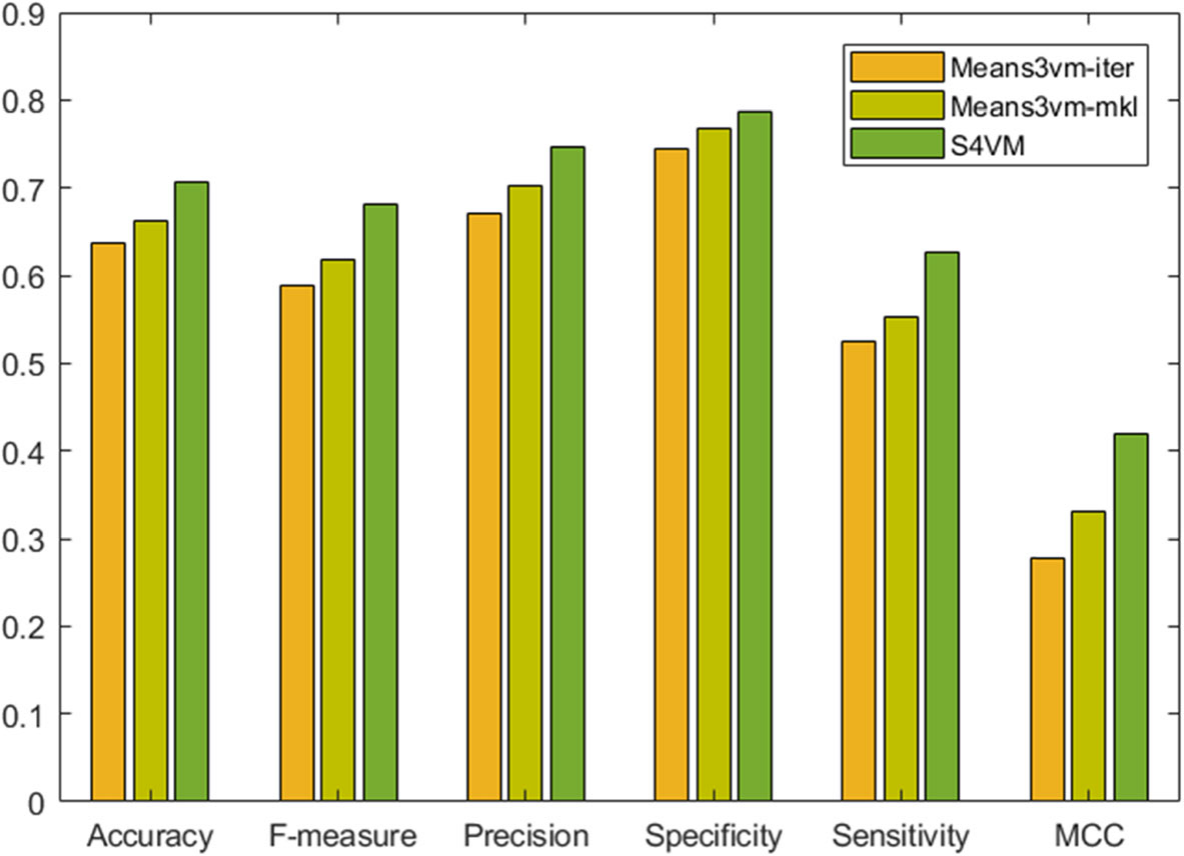
Classification performance evaluation of three Semi-supervised methods on datasets

It can be seen from Fig. 1 that the proposed three semi-supervised methods can classify the protein interaction and non-interaction sites on protein sequences. Means3vm-iter predictor can get good prediction measures, i.e., 0.636 of accuracy, 0.589 of F-measure, 0.67 of precision, 0.745 of specificity, 0.526 of sensitivity, and 0.278 of MCC, from the original protein residue data set D. Compared to Means3vm-iter method, the Means3vm-mkl based-model shows better prediction performance, where the accuracy rate and F-measure are increased by 3%. The reason is that multi-core learning can utilizes the feature mapping capabilities of each basic kernel, and the data is better expressed in the combined feature space constructed by multiple feature spaces, which can significantly improve the classification accuracy. But the process of multi-core learning is complex, and the time required is relatively longer than the method based on iterative optimization. Means3vm-iter transforms the optimization problem into a quadratic programming which and is, thus, easily and quickly solved by standard programs, although it may fall into local minimum, and the classification accuracy is slightly lower than the Means3vm-mkl based-model.

It also can be found that the S4VM method can achieve the best prediction performance among the three semi-supervised models in all of the seven predictor measures, i.e., 0.707 of accuracy, 0.787 of specificity, 0.627 of sensitivity, 0.746 of precision, 0.681 of F measure and 0.419 of MCC. The overall prediction performance of S4VM is improved by more than 4% compared to Means3vm-iter and Means3vm-mkl methods. S4VM attempts to consider all possible low-density boundaries to effectively prevent performance degradation, and therefore it can deduce the false negative rate and false positive rate in prediction, which can be confirmed by the relatively high values of 0.787 and 0.627 for specificity and sensitivity, respectively.

**Fig. 2:**
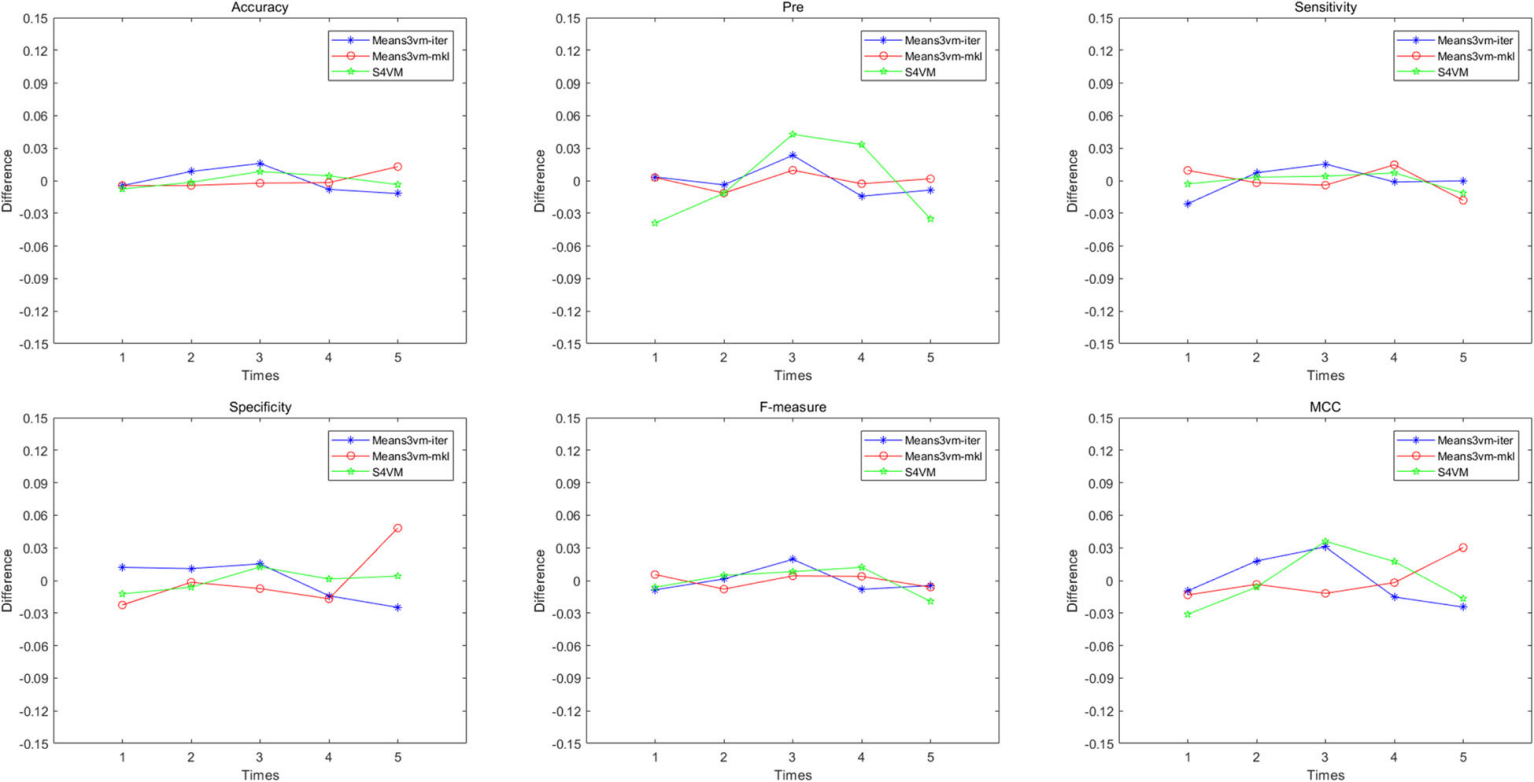
Prediction performance measures in 5 repetitions of cross-validation

To assess the generalization ability of prediction models, a five cross-validation strategy is adopted within the predictors’ construction. For each performance indicator, the differences between its value in each run and mean value in all of five cross-validation times are calculated. It can be observed from Fig. 2 that the three semi-supervised algorithms-based predictors are robust, and most of their fluctuation range is less than 0.03, which indicates that the proposed models have excellent generalization ability when new samples are introduced. Among them, the biggest difference of accuracy is Means3vm-iter model which has value of 0.015, precision in S4VM model with 0.043, sensitivity in Means3vm-iter model with 0.021, F-measure in Means3vm-iter model with 0.02, MCC in S4VM model with 0.036 and only Means3vm-mkl has a specificity of 0.048. Among the three predictors, S4VM performs best, and the mean value of difference is only 0.005 for accuracy, 0.032 for precision, 0.006 for sensitivity, 0.007 for specificity, 0.01 for F-measure and 0.021 for MCC.

## Discussion

It can be found that the three semi-supervised models proposed in this work can predict protein-protein interaction sites based on the features. To further evaluate the effectiveness of these models, the results of some previous works had been used to compare prediction performance.

### Prediction performance comparison between supervised and semi-supervised SVM

Most studies adopted supervised machine learning algorithms to predict interaction sites from protein sequences or structures in previous works, and some of them have to use data sampling technologies to balance the number of positives and negatives to void the prediction bias [27–30]. Instead of semi-supervised methods where all of samples in model training are labeled, semi-supervised machine learning approaches are trying to learning from labeled and unlabeled samples, which can effectively make use more information for learning, which is very significant for the studies of protein interaction where many of them are still unknown.

**Fig. 3:**
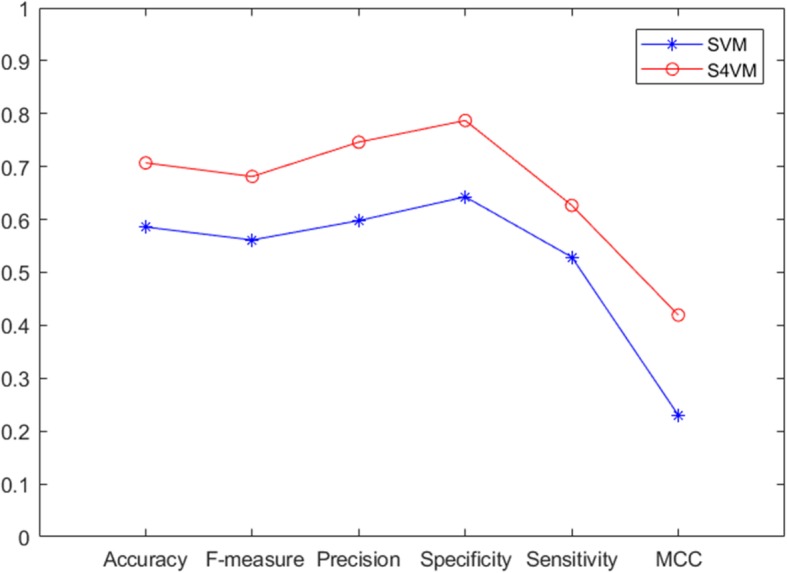
Comparison of experimental results between SVM and S4VM

To evaluate the value of unlabeled sample information in protein interface residues, the traditional supervised SVM algorithm is also directly used to make predictions of protein interaction sites, and its result is shown in Fig. 3. Based on the same dataset, it can be seen that the predictive performance of supervised SVM-based predictor is much lower than that of semi-supervised based one, and the accuracy is only 0.586, which is 0.12 lower than that of S4VM. On other measures, the proposed semi-supervised models also outperform the supervised predictor, i.e., the F-measure is only 0.56, MCC is only0.23, precision is only 0.598, sensitivity is only 0.529 and specificity is only 0.643.These results suggest that unlabeled sample information, when used in conjunction with a small data set of labeled data, can get much improvement in learning accuracy, which is important for the current situation that many protein interactions are not identified by experiments.

### Comparison with other approaches

**Fig. 4:**
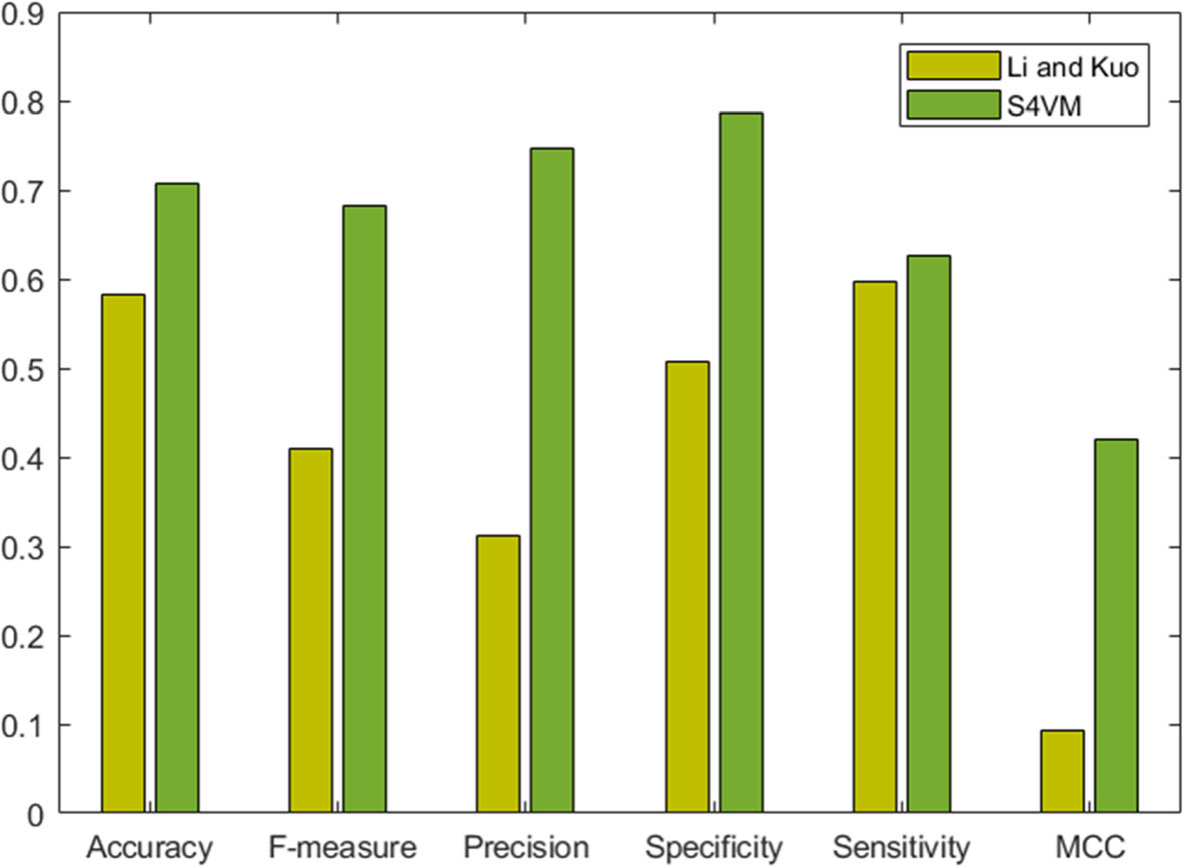
Compared with the former’s evaluation performance

In this work, five features related to amino acid conservation are extracted from protein sequence for identification of protein interface residues from protein surface. To validate the effectiveness of the extracted features in discrimination between interface and non-interface residues, the comparison with a previous study based on the same data set by Li and Kuo has been taken. Compared to the evolutionary conservation features used in this work, they predicted protein interaction sites using five sequence features. Figure 4 shows that both evolutionary and sequence features can successfully identify protein interaction sites, but evolutionarily conserved features show stronger classification capacity than that of sequence features. It can be found that our proposed method can produce more accurate prediction than sequence features-based model did, i.e., 0.124 higher in accuracy, 0.03 in sensitivity, 0.279 in specificity, 0.272 in F-measure and 0.326 in MCC. Especially, the value of precision measure has a 0.435 higher than that in Li and Kuo’s work, which means the false positive rate of prediction deduced dramatically, and the features in this work are really sound in discrimination between protein interaction and non-interaction sites.

### Visualization of experimental results

**Fig. 5:**
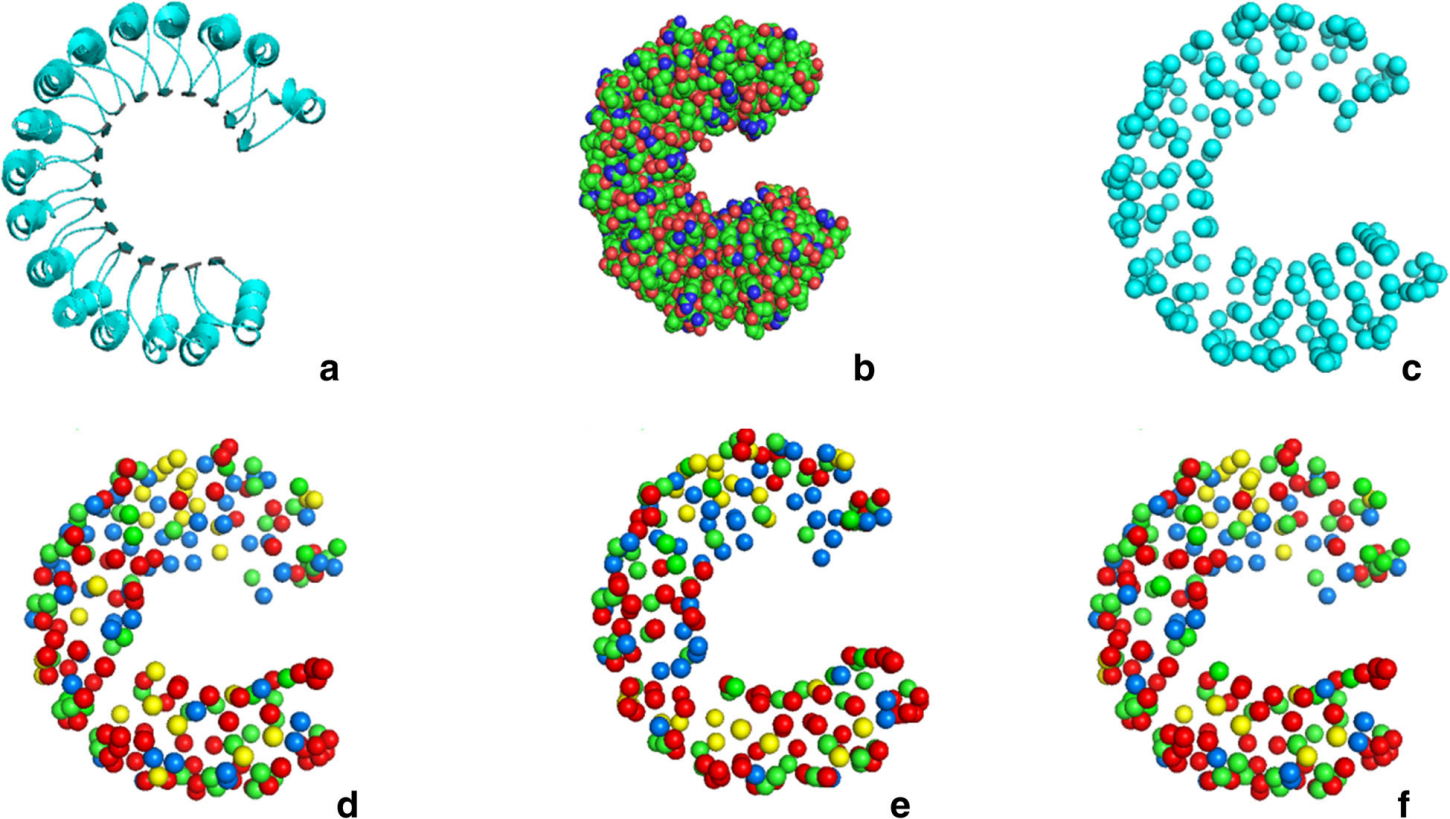
Experimental visualization results. **a** represents the protein chain 1A4Y_A(**a** and **b**) is its spherical representation. **c** is the 1A4Y protein chain after extraction of surface residues. **d**, **e** and **f** show the predicted results of Means3vm-mkl, Means3vm-iter and S4VM, and the green balls, red balls, yellow balls and blue balls represent the number of TP, TN, FP and FN, respectively

To further validate the predictions achieved by our proposed semi-supervised model, a test on one chain of protein complex 1A4Y was taken as an example. We use the molecular visualization tool - Pymol to show our predictions. Figure 5 shows the protein chain 1A4Y_A data set and the results obtained under three semi-supervised models. In D, E, and F, there are 218 balls that represent the surface residues involved in the prediction. Green balls, red balls, yellow balls and blue balls represent the number of TP, TN, FP and FN, respectively. The numbers in details on the complex 1A4Y can be found in Table 1. Our approach improves overall predictive performance and reduces false positives, and S4VM methods perform well. Only 5.2% of the interface residue in the S4VM method was not predicted.

**Table 1:** The number of predictions in TP, TN, FP and FN

	Samples	Results			
		TP	TN	FP	FN
Means3vm-iter	218	57	82	28	51
Means3vm-mkl		60	84	26	48
S4VM		68	87	23	40

## Conclusion

This paper proposed a semi-supervised learning strategy for protein interaction site prediction. Firstly, a non-redundancy dataset with 91 protein chains were selected, and five evolutionary conserved features were extracted for the vectorization of each amino acid residue from the common databases and servers. Then three semi-supervised learning methods, Means3vm-mkl, Means3vm-iter and S4VM are proposed to identity interaction sites from surfaces of protein complexes. The experimental results show that the Means3vm-mkl and S4VM methods have excellent classification results a five-fold cross-validation is used, and the accuracy is 0.662 and 0.707, respectively. The S4VM method can achieve the best overall performance, such as the highest value 0.419 for MCC and 0.681 for F-measured value. By comparison with supervised model and other studies, this work produced much improvement in protein interaction sites prediction, which suggests that the effectiveness of the proposed semi-supervised strategies in discrimination of protein interaction and non-interaction sites. Furthermore, the experimental results demonstrated that residues in protein surface, even its interface label cannot be tagged yet, contain a lot of information of protein interactions, which is important for understanding cellular activity and drug design.

## Methods

### Dataset

The dataset used in this work are come from a previous work investigated by Ansari and Helms et al., where 170 pairs of transient protein interactions has been collected [31]. Protein chains of less than 50 residues and some outdated small family protein chains are discarded to make the data more representative. If there is a plurality of interacting partners for the same chain, the partner chain with most interfacial residues is represented. The BLASTCLUST program was used to exclude protein chains with sequence similarity greater than 30%, and finally 91 non-redundant protein chains has been remained for this study, which can be found in Table 2 [15, 32, 33].

**Table 2:** The protein chains used in this work

1AY7_A	1B6C_A	1B7Y_B	1AZS_B	1B7Y_A	1AVG_H	1AZS_C	1B6C_B
1UDI_E	1UGH_E	1ZBD_A	1UEA_A	1UUZ_A	1TCO_A	3TGI_I	1WQ1_G
1HLU_P	1IRA_Y	1KKL_A	1HWH_B	1JSU_C	1HLU_A	1IRA_X	1ITB_A
1BDJ_B	1BMQ_A	1BRB_I	1BGX_T	1BP3_A	1BDJ_A	1BI7_A	1BMQ_B
1QBK_B	1SMP_A	7CEI_A	1QBK_C	1STF_E	1PYT_B	1SGP_E	1SMP_I
1FLT_Y	1GLA_F	1HJA_C	1GFW_A	4SGB_I	1FLT_V	1GFW_B	1GLA_G
1ABR_A	1AHW_C	1ATN_D	1ABR_B	1AK4_D	1A4Y_A	1ACB_I	1AK4_A
1BVK_A	1CA0_B	1D4V_B	1BVK_C	1D4V_A	1BRS_A	1BVN_P	1CXZ_B
2KAI_B	2SIC_I	3SGB_I	2PCC_A	2TEC_E	1ZBD_B	2PCC_B	2SNI_I
1DAN_U	1E9H_B	1FAP_B	1DFJ_E	1ETH_A	1DAN_L	1E96_A	1EFU_B
1L0Y_A	1NOC_B	1PYT_A	1L0Y_B	1PDK_B	1KKL_H	1MAH_A	1PDK_A
1GUA_B	1STF_I	1UEA_B					

The definitions of the residues are same as what Fariselli et al. did in their work [30]. Surface residues are defined if the relative accessible surface area residue is bigger than 16% of the maximum accessible surface area for each type of amino acid. Among the surface residues, a residue can be defined as interface residues if the distance between its alpha carbon atom and that of any residues in the interaction chain is less than 1.2 nm, otherwise it will be categorized as non-interface residues. Based on the above definitions, the original residue data set D is composed of 2299 interface residues and 8131 non-interface residues which are obtained from the 91 protein chains used in this work.

### Feature extraction

There are many properties of amino acids had been used for protein interactions or interaction sites prediction, among them evolutionary conservation analyses have been widely applied to characterize functionally/structurally important residues because these amino acids in a protein sequence are conserved through selective evolutionary pressure [20, 34–36]. In this work, five evolutionary conservation relevant features are extracted for protein interaction sites prediction, where residue spatial sequence, sequence information entropy, relative entropy and residue sequence weight are extracted from the HSSP database, and evolutionary rate residues are extracted from Consurf Serve.

The spatial sequence profile of amino acid residues, a feature widely used in protein related studies, represents the frequency of various amino acids at a given residue position in the primary structure of proteins. Protein residue sequence entropy is based on Shannon’s information theory to estimate the conservation score of sequence variability. Relative entropy is the normalized sequence information entropy. The conserved weight of the residue sequence is a calculation of position conservativeness of the protein sequence. The evolution rate of residues can be traded off from a statistical point of view, considering the linkages generated by the system in the stochastic process of sequence and evolution and the maximum likelihood estimate of the evolution rate can be accessed using the Rate4Site algorithm to calculate the conservation of each amino acid position [37]. For each residue, 20 dimensions for protein sequence profile and one dimension for each of other four features can be extracted in this work.

As many previous studies did, a slide-window strategy is also adopted in this work to consider the interface information, which is formed by the target residues centered with 10 spatially closest ones. Therefore, each target residue can be represented by a 264-dimensional vector and used for subsequent prediction construction.

### Semi-supervised models

**Fig. 6:**
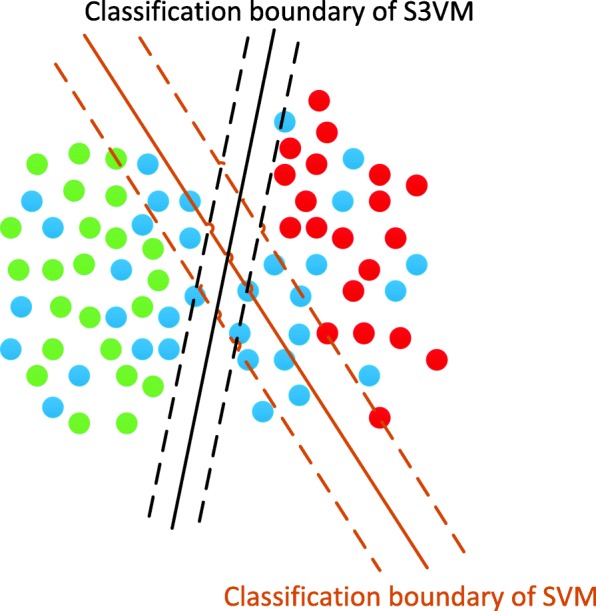
Illustration of different classification boundaries of SVM which considers only labeled data, and S3VM which considers labeled and unlabeled data, where green balls denote positives, red ones are negatives, and blue ones are unlabeled samples

In semi-supervised learning, the labeled sample set is {*x*_1_, …, *x*_*l*_}{x_1_, …, x_l_}, and the unlabeled sample set is {*x*_*l* + 1_, …, *x*_*l* + *u*_}, where l and u are the number of labeled and unlabeled samples, respectively, *y*_*i*_ = {+1, −1}. The labels of labeled and unlabeled sample set can noted as *I*_*l*_ = {1, …, *l*}, and *I*_*u*_ = {*l* + 1, *l* + 2, …, *l* + *u*}. As one of the most popular semi-supervised learning methods, Semi-supervised support vector machines (S3VM) attempts to standardize and adjust decision boundaries by exploring unlabeled data based on clustering assumptions, whose illustration can be found in Fig. 6 [26, 38]. The meanS3VM, a fast S3VM algorithm, estimates the category average of the unlabeled data, so that the classification performance is very similar to the supervised SVM. The core idea of meanS3VM algorithm is to maximize the interval between the class averages of the two categories of samples, and thus the goal is to find the decision function *f*(*x*) = *w*^′^ ∅ (*x*) + *b* to minimize.

1 $$ \underset{d\in \Delta  }{\mathit{\min}}\underset{w,b,\rho, \xi }{\mathit{\min}}\frac{1}{2}{\left\Vert w\right\Vert}^2+{c}_1{\sum}_{i=1}^l{\xi}_i-{c}_2\rho $$

$$ s.t.{y}_i\left({w}^{\prime}\varnothing \left({x}_i\right)+b\right)\ge 1-{\xi}_i,{\xi}_i\ge 0,i=1,\dots, l, $$

$$ \frac{1}{u_{+}}\left({w}^{\prime}\sum \limits_{j=l+1}^{l+u}{d}_{j-l}\varnothing \left({x}_j\right)\right)+b\ge \rho $$

$$ \frac{1}{u_{-}}\left({w}^{\prime}\sum \limits_{j=l+1}^{l+u}\left(1-{d}_{j-l}\right)\varnothing \left({x}_j\right)\right)+b\le -\rho $$

$$ \sum \limits_{i\in {I}_u}\mathit{\operatorname{sgn}}\left({w}^{\prime}\varnothing \left({x}_i\right)+b\right)=r $$

Herein, the last constraint is an equilibrium constraint that avoids assigning all unlabeled samples to the same category, *r* is a user-defined parameter, and $\Delta  =\left\{d|{d}_i\in \left\{0,1\right\},\sum \limits_{i=1}^u{d}_i={u}_{+}\right\}$*,* ${u}_{+}=\frac{r+u}{2}$*,* ${u}_{-}=\frac{-r+u}{2}$. Since the bilinear constrains between *w* and *b*, this formula is non-convex, and the two algorithms can be used to solve it. The first one is based on multiple kernels learning (MeanS3VM_mkl), while the second one is based on alternating optimization (MeanS3VM_iter) [24].

1. MeanS3VM_mkl

Mathematically, the goal of S3VM can be solved with the dual form:

2 $$ mi{n}_{d\in \varDelta } ma{x}_{\alpha \in A}{\alpha}^{\hbox{'}}\tilde{l}-\frac{1}{2}{\left(\alpha \bullet \tilde{y}\right)}^{\hbox{'}}{K}^d\left(\alpha \bullet \tilde{y}\right) $$

which can be expressed in the form of a multicore learning optimization problem:

3 $$ mi{n}_{\mu \in M} ma{x}_{\alpha \in A}{\alpha}^{\hbox{'}}\tilde{I}-\frac{1}{2}{\left(\alpha \bullet \tilde{y}\right)}^{\hbox{'}}\left({\varSigma}_{t: dt\in \varDelta}\mu t{K}^{dt}\right)\left(\alpha \bullet \tilde{y}\right) $$

where *M* = {*μ*| ∑*u*_*t*_ = 1, *u*_*t*_ ≥ 0 },

$$ \alpha ={\left[{\alpha}_1,\dots, {\alpha}_{l+2}\right]}^{\prime}\epsilon {R}^{l+2} $$

$$ \overset{\sim }{I}={\left[{I}_1,\dots, {I}_t,0,0\right]}^{\prime}\in {R}^{l+2} $$

$$ \overset{\sim }{y}={\left[{y}_1,\dots, {y}_t,1,-1\right]}^{\prime}\in {R}^{l+2} $$

$$ A=\Big\{\alpha \mid \sum \limits_{i=1}^{l+2}{\alpha}_i\overset{\sim }{y_i}=0,\sum \limits_{i=1}^{l+2}{\alpha}_i={c}_2;0\le {\alpha}_i\le {c}_1, $$

$$ \forall i=1,\dots, l;0\le {\alpha}_l+1,{\alpha}_l+2\le {c}_2\Big\} $$

The nuclear matrix *K*^*d*^ ∈ *R*^(*l* + 2) ∗ (*l* + 2)^, the element is ${K}_{ij}^d={\left({\varnothing}_i^d\right)}^{\prime}\left({\varnothing}_i^d\right)$.

4 $$ {\varnothing}_i^d=\left\{\begin{array}{c}\frac{1}{u_{+}}\sum \limits_{j=l+1}^{l+u}{d}_j-l\varnothing \left({x}_i\right)i=l+1\\ {}\varnothing \left({x}_i\right)i=1,\dots, l\\ {}\frac{1}{u_{-}}\sum \limits_{-j=l+1}^{l+u}\left(1-{d}_j-l\right)\varnothing \left({x}_i\right)\ i=l+2\end{array}\ \right. $$

Since all *d*_*t*_ ∈ *∆* are to be minimized and there will be a large number of reasonable *d*_*t*_, the cut plane algorithm is adopted to solve the above problem and find the optimal label *d* vector of the unlabeled samples, whose details can be found in references [17, 24].

2) *Means3vm-iter*

Another way to solve the problem of MeanS3VM is to alternate optimization, which can be abbreviated as:

5 $$ \underset{d\in \Delta , \rho }{\mathit{\max}}\rho $$

$$ s.t.\frac{1}{u_{+}}\left({w}^{\prime}\sum \limits_{j=l+1}^{l+u}{d}_j-l\varnothing \left({x}_j\right)\right)+b\ge \rho $$

$$ \frac{1}{u_{-}}\left({w}^{\prime}\sum \limits_{j=l+1}^{l+u}\left(1-{d}_j-l\right)\varnothing \left({x}_j\right)\right)+b\le -\rho $$

In the optimization process, if *f*(*x*_*i*_) > *f*(*x*_*j*_)*,* ∀*i*, *j* ∈ *I*_*u*_, then *d*_*i* − 1_ ≥ *d*_*j* − 1_, which has been proved [11]. Assigning labels to unlabeled samples based on predicted values using this theorem, which can ensure that the label *d* vector obtained each time is better than the previous one [17, 24].

### S4VM

Given a large amount of unlabeled samples in data set, there may be multiple “intervals” of low-density boundaries, and it is difficult to determine which one is the best. Although these low-density boundaries and the number of labeled samples are limited, due to the large differences, there will be a large loss if the selection is wrong, resulting in performance degradation, even worse than using only labeled samples, which limits the use of semi-supervised learning methods in certain key areas.

S4VM has been improved on traditional S3VM. The difference between S4VM and S3VM is that S3VM tries to focus on the best low-density boundary, while S4VM focuses on multiple possible low-density boundaries. The main idea is to optimize without giving many different “interval” boundaries. Class division of labeled samples. This maximizes the performance improvement of the support vector machine over the worst case labeled samples. The specific practices are as follows:

$\mathrm{h}\left(\mathrm{f},\hat{\mathrm{y}}\right)$ is the objective function to be optimized by S3VM.

6 $$ h\left(f,\hat{y}\right)=\frac{{\left\Vert f\right\Vert}_H}{2}+{C}_1{\sum}_{i=1}^ll\left({y}_i,f\left({x}_i\right)\right)+{C}_2{\sum}_{j=1}^ul\left(\hat{y_j},f\left(\hat{x_j}\right)\right) $$

The goal is to find multiple low-density boundary lines ${\left\{{\mathrm{f}}_{\mathrm{t}}\right\}}_{\mathrm{t}=1}^{\mathrm{T}}$ with “intervals” and the corresponding category division ${\left\{\hat{{\mathrm{y}}_{\mathrm{t}}}\right\}}_{\mathrm{t}=1}^{\mathrm{T}}$ so that the following functions are minimized.

7 $$ \underset{{\left\{{f}_t,\hat{y_t\in \beta}\right\}}_{t=1}^T}{\mathit{\min}}{\sum}_{t=1}^Th\left({f}_t,\hat{y_t}\right)+ M\varOmega \left({\left\{\hat{y_t}\right\}}_{t=1}^T\right) $$

where T is the number of dividing lines, and Ω is a penalty function that measures the differentiation of the dividing line. Various functions can be used in the implementation. M is a large constant used to ensure the difference. Obviously, minimizing (7) can ensure the difference of the boundary line and the large interval. Without loss of generality, we assume that f is a linear function, f(x) = w^′^ ∅ (x) + b. The optimization problem that needs to be solved is expressed as.

8 $$ \underset{{\left\{{w}_t,\hat{b_t,{y}_t\in \beta}\right\}}_{t=1}^T}{\mathit{\min}}{\sum}_{t=1}^T\left(\frac{1}{2}{\left\Vert {w}_t\right\Vert}^2+{c}_1{\sum}_{i=1}^l{\xi}_i+{c}_2{\sum}_{j=1}^u\hat{\xi_j}\right)+ M\varOmega \left({\left\{\hat{y_t}\right\}}_{t=1}^T\right) $$

$$ s.t.{y}_i\left({w}_t^{\prime}\varnothing \left({x}_i\right)+{b}_t\right)\ge 1-{\xi}_i,{\xi}_i\ge 0 $$

$$ \hat{y_{t,j}}\left({w}_t^{\prime}\varnothing \left(\hat{x_j}\right)+{b}_t\right)\ge 1-\hat{\xi_j},\hat{\xi_j}\ge 0 $$

$$ \forall i=1,\dots, l,\forall j=1,\dots, u,\forall t=1,\dots, T $$

Then use an efficient sampling search strategy to solve (8). First, through the local search, find multiple large margin low-density separators. The k-means clustering algorithm is then used to identify representative splitters with a large variety of diversity [16].

### Evaluation criteria

In addition to the accuracy, precision, sensitivity, and specificity which often used to evaluate predicted performance, F-measure and Mathew’s Correlation Coefficient (MCC) values are introduced. F-measure is a weighted harmonic average of recalls and precision, often used to evaluate classification models, and MCC is an effective measure in imbalanced data classification.

11 $$ Accuracy=\frac{TP+ TN}{TP+ FP+ TN+ FN} $$

12 $$ Sensitivity=\frac{TP}{TP+ FN} $$

13 $$ \mathrm{P} recision=\frac{TP}{TP+ FP} $$

14 $$ Specificity=\frac{TN}{FP+ TN} $$

15 $$ F- measure=2\times \frac{Precision\times Sensitivity}{Precision+ Sensitivity} $$

16 $$ MCC=\frac{\left( TP\times TN- FP\times FN\right)}{\sqrt{\left( TP+ FP\right)\times \left( TP+ FN\right)\times \left( TN+ FP\right)\times \left( TN+ FN\right)}} $$

where TP, FP, TN and FN represent the number of true positives (correctly predicted interface residues), the number of false positives (incorrectly predicted interface residues), the number of true negatives (correctly predicted non- interface residues) and the number of false negatives (incorrectly predicted non- interface residues), respectively.

## Abbreviations

HSSP: Homology-derived Secondary Structure of Proteins
MCC: Mathew’s Correlation Coefficient
PPI: Protein-protein interaction
S3VM: Semi-supervised support vector machine
S4VM: Safe semi-supervised support vector machine
